# Multiculturalism, Integration, and Mental Well-being in the Context of Emergency Departments: a Comparative Analysis Across Three Hospitals in Northern Italy

**DOI:** 10.1192/j.eurpsy.2025.1455

**Published:** 2025-08-26

**Authors:** E. Gambaro, M. Prelati, R. Valsesia, C. M. Gramaglia, P. Zeppegno

**Affiliations:** 1 SC Psichiatria, AOU Maggiore della Carità di Novara; 2 Medicina Traslazione, Università del Piemonte Orientale, Novara; 3 SPDC, Azienda Ospedaliero - Universitaria SS. Antonio e Biagio e Cesare Arrigo, Alessandria; 4 SPDC, Ospedale Santissima Trinità, Borgomanero, Italy

## Abstract

**Introduction:**

A migrant is any person who moves or has moved across an international border regardless of the legal status of the person, whether the movement is voluntary or not, the reasons for the movement or the length of stay. In recent decades, migratory flows have increased significantly also in Italy. The migration process has highlighted the need to explore the potential challenges within the local multicultural context, as well as the difficulties that migrant patients with mental health issues may face. The assistance, diagnosis, and treatment of patients of foreign origin have become essential aspects of the pursuit of overall well-being within the community.

According to the literature, migration can represent a stress factor that leads to an increased risk of developing psychiatric symptoms or disorders. It should be emphasized that, although the organization of mental health services in Italy is strongly based on territorial outpatient services, the first contact of migrants with psychiatry occurs through the emergency room and the psychiatric wards of hospitals that usually admit patients with disorders in the acute phase.

It becomes necessary to learn more about this topic, given the limited or insufficient national and international studies on these issues.

**Objectives:**

This study aims to investigate and describe the characteristics of migrants admitted to three Emergency and Acceptance Departments (DEA) in Northern Italy: Novara, Alessandria, and Borgomanero in comparison with natives, to investigate any differences between the two populations regarding the variables described above.

**Methods:**

Through an electronic data acquisition research software solution (REDCap), the clinical and socio-demographic characteristics of migrants hospitalized at the three DEA will be collected. The data of migrants who received psychiatric consultation (PC) from January 2020 to December 2024 will be compared with those of natives. Data will include socio-demographic and clinical aspects, with a specific focus on mental health disorders (such as suicidal behaviours), type of intervention and outcome of the consultation. The only inclusion criterion used was the presence of PC after triage while no exclusion criteria were used except for age under 16.

**Results:**

Ongoing analyzes will provide an in-depth description of the correlation between migrant status and the mental well-being of patients accessing the DEA who have received a PC. Particularly, the analyzes will allow us to outline the risk factors for psychiatric diagnosis and the choice of treatment strategies.

**Conclusions:**

The data could suggest the need for prevention and intervention programs aimed at the migrant population, oriented towards cultural differences in the expression of discomfort, linguistic difficulties and/or mutual understanding between healthcare personnel and patients.

**Disclosure of Interest:**

None Declared

